# Comparison of Four Albumin-Based Liver Reserve Models (ALBI/EZ-ALBI/PALBI/PAL) against MELD for Patients with Hepatocellular Carcinoma Undergoing Transarterial Chemoembolization

**DOI:** 10.3390/cancers15071925

**Published:** 2023-03-23

**Authors:** Shu-Yein Ho, Po-Hong Liu, Chia-Yang Hsu, Yi-Hsiang Huang, Jia-I Liao, Chien-Wei Su, Ming-Chih Hou, Teh-Ia Huo

**Affiliations:** 1 Division of Gastroenterology and Hepatology, Min-Sheng General Hospital, Taoyuan 33044, Taiwan; 2 Department of Medical Research, Taipei Veterans General Hospital, Taipei 11217, Taiwan; 3 School of Medicine, National Yang Ming Chiao Tung University, Taipei 112304, Taiwan; 4 Department of Internal Medicine, University of Texas Southwestern Medical Center, Dallas, TX 75390, USA; 5 Department of Medicine, Renown Medical Center, Reno, NV 89502, USA; 6 Division of Gastroenterology and Hepatology, Department of Medicine, Taipei Veterans General Hospital, Taipei 11217, Taiwan; 7 Institute of Clinical Medicine, National Yang Ming Chiao Tung University, Taipei 112304, Taiwan; 8 Institute of Pharmacology, National Yang Ming Chiao Tung University, Taipei 112304, Taiwan

**Keywords:** hepatocellular carcinoma, ALBI, EZ-ALBI, PALBI, PAL, MELD

## Abstract

**Simple Summary:**

Liver functional reserve plays a critical role in the management of hepatocellular carcinoma (HCC) patients. The model for end-stage liver disease (MELD) is used to assess liver functional reserve in HCC patients. Alternatively, the albumin-bilirubin (ALBI) score and easy (EZ)-ALBI score, platelet-albumin-bilirubin (PALBI) score and platelet-albumin (PAL) score are also proposed to indicate liver reserve in HCC. We aimed to compare these four albumin-based liver reserve models (ALBI/EZ-ALBI/PALBI/PAL) against MELD for HCC patients undergoing transarterial chemoembolization (TACE). Among these models, the PALBI grade had the highest homogeneity and lowest corrected Akaike information criteria value, followed by EZ-ALBI, PAL, ALBI and lastly, MELD. All four albumin-based liver reserve models are better prognostic tools than MELD scores in patients undergoing TACE. Of these, the PALBI score is the best model to evaluate the liver reserve and should be considered a surrogate marker in these patients.

**Abstract:**

(1) Background: The severity of liver functional reserve plays an important role in the management of hepatocellular carcinoma (HCC). Noninvasive models such as the model for end-stage liver disease (MELD), albumin-bilirubin (ALBI) grade and easy (EZ)-ALBI grade, platelet-albumin-bilirubin (PALBI) and platelet-albumin (PAL) are used to evaluate liver dysfunction. We aimed to compare the prognostic performance of these four albumin-based models against MELD in HCC patients undergoing transarterial chemoembolization (TACE). (2) Methods: A total of 1038 treatment naïve HCC patients who received TACE as the primary treatment were enrolled. A multivariate Cox model was used to determine independent survival predictors. (3) Results: Multivariate analysis revealed that higher serum creatinine and α-fetoprotein level, vascular invasion, large tumor size, ALBI grades 2–3, EZ-ALBI grades 2–3, PALBI grades 2–3, PAL grades 2–3, but not the MELD score, were independent predictors associated with decreased survival in different Cox models. Among these models, the PALBI grade had the highest homogeneity and lowest corrected Akaike information criteria value, followed by EZ-ALBI, PAL, ALBI and, lastly, MELD. (4) Conclusions: All four albumin-based liver reserve models are better prognostic tools than MELD score in HCC patients undergoing TACE. Of these, the PALBI score is the best model to evaluate the liver reserve and should be considered a surrogate marker in these patients.

## 1. Introduction

Hepatocellular carcinoma (HCC) is the fourth leading cause of cancer-related death globally [1]. Its main risk factors are chronic hepatitis B and C virus (HBV, HCV) infections, alcoholism and non-alcoholic fatty liver disease (NAFLD) [2,3]. The prognosis of HCC is usually poor because many patients are diagnosed at an advanced stage. According to current HCC practice guidelines [4,5], surgical resection, local ablation and transplantation are recommended for very early and early-stage HCC. Patients at intermediate or advanced stages are suggested to receive transarterial chemoembolization (TACE) or systemic therapy, including targeted- and immuno-therapy [6,7].

TACE is a safe, effective and usually the first-line treatment to prolong survival in patients with multinodular HCC unsuitable for curative treatments [8,9,10,11]. For these patients, the severity of liver dysfunction is tightly linked with their long-term outcome. The Child–Turcotte–Pugh (CTP) score is a commonly used tool to evaluate liver injury [12]. However, the CTP score has drawbacks due to inter-related objective variables and subjective variables such as ascites and hepatic encephalopathy. The model for end-stage liver disease (MELD) was shown to be a better alternative method initially used in selecting patients for timely liver transplantation [12,13]; however, it also harbors the disadvantage of very complex calculation.

The albumin-bilirubin (ALBI) score, based solely on the serum albumin and bilirubin levels, is an objective marker of the liver functional reserve [14,15,16,17]. The easy (EZ)-ALBI score is an updated version of the ALBI with adequate predictive accuracy [18,19,20]. A potential shortcoming of the ALBI and EZ-ALBI grades is that they do not include any parameters to indicate portal hypertension in liver cirrhosis. Therefore, the platelet-albumin-bilirubin (PALBI) score, which includes a platelet count, a surrogate marker in portal hypertension, was proposed to evaluate liver reserve in HCC [21,22,23,24,25,26]. More recently, a simplified version, the platelet-albumin (PAL) score, was developed to assess short- and long-term survival in HCC patients receiving surgical resection [27,28]. In this study, we specifically compared the prognostic performance of these four albumin-based liver reserve models (ALBI, EZ-ALBI, PALBI and PAL) against MELD in HCC patients undergoing TACE.

## 2. Materials and Methods

### 2.1. Patients

Over a 16-year period between 2002 and 2018, a total of 1038 treatment-naïve HCC patients receiving TACE as their primary treatment at Taipei Veterans General Hospital were enrolled. Their clinical information, including baseline demographics, the extent of tumor involvement (number and size of the tumor, vascular invasion and metastasis), laboratory data, the severity of liver functional reserve (CTP class, ALBI grade, EZ-ALBI grade, PALBI grade, PAL grade and MELD score) and cancer staging, were recorded at the time of diagnosis. This study was approved by the Institutional Review Board of the Taipei Veterans General Hospital and complies with current ethical guidelines.

### 2.2. Diagnosis and Definition

The diagnosis of HCC was based on typical radiological characteristics (early arterial enhancement in the arterial phase and delayed wash-out in the venous phase) by contrast-enhanced computed tomography (CT) scan or magnetic resonance imaging (MRI) [4,5]. The performance status (PS) was assessed by the Eastern Cooperative Oncology Group (ECOG) criteria [29]. The calculation and grading of the liver reserve models (MELD, ALBI, EZ-ALBI, PALBI and PAL) are summarized in Table 1 [14,18,25,27,30].

### 2.3. Treatments

The inclusion criteria of TACE were: (1) unresectable HCC or patients not eligible or unwilling to receive other therapies; (2) CTP class A or B; (3) no main portal vein thrombosis; (4) serum creatinine level < 1.5 mg/dL and (5) no gross ascites by sonography or CT scan [31]. Treatment selection was discussed at the multidisciplinary HCC Board of the Taipei Veterans General Hospital. The information, such as benefits and risks, was provided to the individual patient based on shared decision making. Written informed consent was obtained prior to initiation of the treatment. The details of the TACE procedure were described in our previous studies [32,33]. After TACE, patients were followed up every 2–3 months for serum α-fetoprotein (AFP) level, ultrasonography, CT or MRI to confirm therapeutic response and further treatment planning.

### 2.4. Statistics

Statistical analyses were performed using IBM SPSS Statistics for Windows, version 25.0 (IBM Corp., Armonk, NY, USA). The Mann–Whitney rank-sum test was used to compare continuous variables; the comparison of categorical data was evaluated by the Pearson chi-squared test or the two-tailed Fisher’s exact test. Overall survival (OS) was estimated using the Kaplan–Meier method and compared with the log-rank test. Independent prognostic predictors associated with survival were assessed by the multivariate Cox proportional hazards model to generate a hazard ratio (HR) and 95% confidence interval (CI). For all tests, a *p* < 0.05 was considered statistically significant. The prognostic performance among different liver reserve models was compared using the corrected Akaike information criteria (AICc) and homogeneity. The lower the AICc, the more explanatory and informative the model is [34,35].

## 3. Results

### 3.1. Patient Characteristics

**Figure 1:**
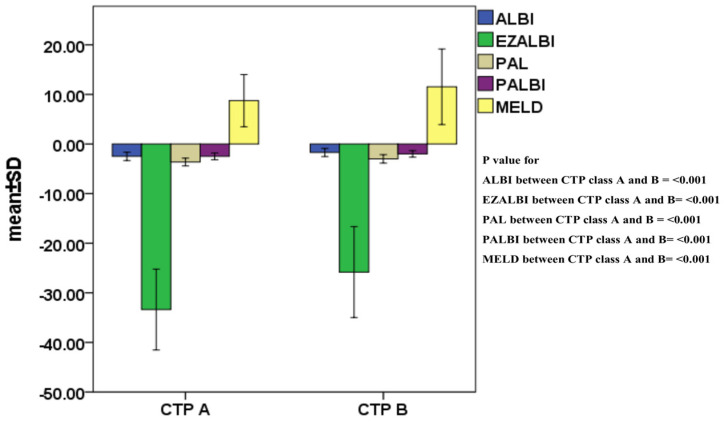
Comparison of the mean scores of ALBI, EZ-ALBI, PALBI, PAL and MELD according to CTP class A vs. B. All the scores in CTP B display a higher (or less negative) value in comparison with the scores in CTP class A (all *p* < 0.001).

The baseline characteristics of patients are summarized in Table 1. Their mean age was 67 years and the majority were male. HBV and HCV were the most common etiologies of HCC. About 53% of patients had a single tumor nodule, and the mean tumor diameter was 6.3 cm. Approximately 79% of patients were CTP classification A and 18% of patients had ascites at the time of diagnosis. The grading and mean values of ALBI, EZ-ALBI, PALBI, PAL and MELD scores are given in Table 2. The mean scores of these models according to CTP class A vs. B are shown in Figure 1. All the scores in CTP B display a higher (or less negative) value in comparison with the scores in CTP class A (all *p* < 0.001).

### 3.2. Survival Analysis

**Figure 2:**
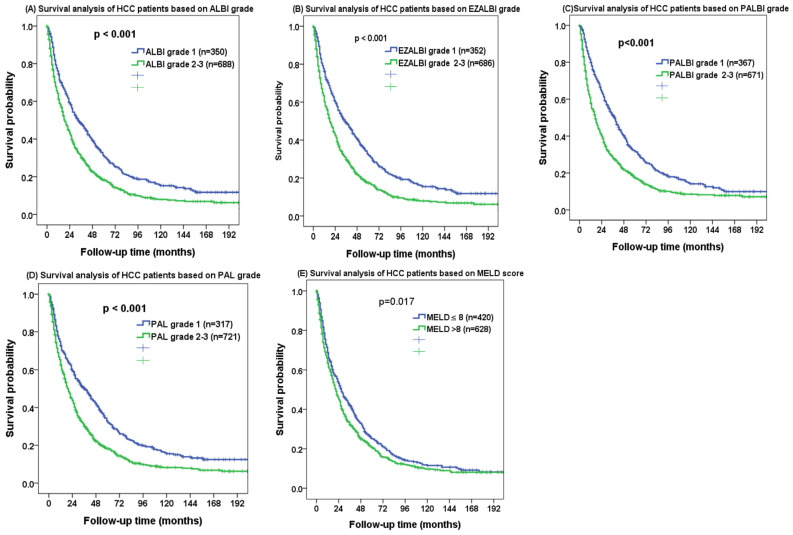
Kaplan–Meier survival analysis of HCC patients undergoing transarterial chemoembolization (TACE) based on (**A**) ALBI grade, (**B**) EZ-ALBI grade, (**C**) PALBI grade, (**D**) PAL grade and (**E**) MELD score.

The mean and median follow-up periods were 35 and 21 months, respectively. The ALBI grade 1 patients had better OS compared with the ALBI grades 2–3 patients. The 1-, 3- and 5-year OS were 74%, 46% and 31% for ALBI grade 1 patients, and 60%, 31% and 18% for ALBI grade 2–3 patients, respectively (Figure 2A, *p* < 0.001). The EZ-ALBI grade 2–3 patients had worse long-term survival compared with grade 1 patients. The 1-, 3- and 5-year OS were 75%, 47% and 32% for EZ-ALBI grade 1 patients, and 60%, 21% and 18% for grade 2–3 patients, respectively (Figure 2B, *p* < 0.001). Consistently, the PALBI grade 1 patients had better OS compared with grade 2–3 patients. The 1-, 3- and 5-year OS were 81%, 51% and 32% for PALBI grade 1 patients and 57%, 18% and 17% for grade 2–3 patients, respectively (Figure 2C, *p* < 0.001). The PAL grade 1 patients had a lower risk of mortality compared with grade 2–3 patients. The 1-, 3- and 5-year OS were 72%, 49% and 31% for PAL grade 1 patients, and 62%, 31% and 19% for grade 2–3 patients, respectively (Figure 2D, *p* < 0.001). Patients with a MELD score of ≤8 had better survival than those with a score of >8. The 1-, 3- and 5-year OS were 71%, 43% and 25% for MELD score ≤ 8 patients and 66%, 33% and 21% for score > 8 patients, respectively (Figure 2E, *p* = 0.017).

### 3.3. Multivariate Cox Analysis

When the ALBI grade was examined along with other variables in the univariate analysis (Table 3), thrombocytopenia, lower serum albumin levels, higher creatinine levels, presence of ascites, alpha-fetoprotein (AFP) level > 200 ng/mL, vascular invasion, larger tumor size, performance status 2 and ALBI grades 2–3 were associated with increased mortality. Multivariate analysis revealed that creatinine (HR: 1.267, *p* = 0.003), AFP >200 ng/mL (HR: 1.745, *p* < 0.001), vascular invasion (HR: 1.621, *p* < 0.001), tumor size > 5 cm (HR: 1.863, *p* < 0.001) and ALBI grades 2–3 (HR: 1.541, *p* < 0.001) were linked with decreased OS.

Using a similar approach in the univariate analysis of patients including the EZ-ALBI grade (Table 4), thrombocytopenia, lower serum albumin levels, higher creatinine levels, ascites, AFP > 200 ng/mL, vascular invasion, larger tumor size, performance status 2 and EZ-ALBI grades 2–3 were associated with decreased long-term survival. Multivariate analysis revealed that serum creatinine (HR: 1.260, *p* = 0.004), AFP > 200 ng/mL (HR: 1.729, *p* < 0.001), vascular invasion (HR: 1.613, *p* < 0.001), tumor size > 5 cm (HR: 1.853, *p* < 0.001) and EZ-ALBI grades 2–3 (HR: 1.563, *p* < 0.001) were linked with a higher risk of mortality.

In the univariate analysis of patients including the PALBI grade (Table 5), thrombocytopenia, lower albumin levels, higher creatinine levels, ascites, AFP > 200 ng/mL, vascular invasion, larger tumor size, performance status 2 and PALBI grades 2–3 were associated with decreased long-term survival. Multivariate analysis revealed that creatinine (HR: 1.338, *p* = 0.001), AFP > 200 ng/mL (HR: 1.694, *p* < 0.001), vascular invasion (HR: 1.596, *p* < 0.001), tumor size > 5 cm (HR: 1.790, *p* < 0.001) and PALBI grades 2–3 (HR: 1.358, *p* < 0.001) were linked with shortened survival.

In the univariate analysis of patients including the PAL grade (Table 6), thrombocytopenia, lower albumin levels, higher creatinine levels, ascites, AFP > 200 ng/mL, vascular invasion, larger tumor size, performance status 2 and PAL grades 2–3 were associated with decreased survival. Multivariate analysis revealed that creatinine (HR: 1.261, *p* = 0.004), AFP > 200 ng/mL (HR: 1.732, *p* < 0.001), vascular invasion (HR: 1.663, *p* < 0.001), tumor size > 5 cm (HR: 1.949, *p* < 0.001) and PAL grades 2–3 (HR: 1.541, *p* < 0.001) predicted increased mortality.

In the univariate analysis of patients including the MELD score (Table 7), thrombocytopenia, lower albumin levels, higher creatinine levels, ascites, AFP > 200 ng/mL, vascular invasion, larger tumor size, performance status 2 and MELD score > 8 were associated with decreased long-term survival. Multivariate analysis revealed that albumin (HR: 1.451, *p* < 0.001), creatinine (HR: 1.270, *p* = 0.003), AFP > 200 ng/mL (HR: 1.709, *p* < 0.001), vascular invasion (HR: 1.673, *p* < 0.001) and tumor size > 5 cm (HR: 1.855, *p* < 0.001) were independent predictors associated with shortened survival.

### 3.4. Prognostic Performance of the Five Non-Invasive Liver Reserve Models

The comparison of the prognostic performance of the five non-invasive liver reserve models is shown in Table 8. Of these, the PALBI grade had the highest homogeneity and lowest AICc value, followed by the EZ-ALBI, PAL, ALBI grades and lastly, the MELD score. Thus, all four albumin-based models had better prognostic performance than the MELD.

## 4. Discussion

The prognosis of HCC depends not only on the extent of the tumor but also on the severity of the liver reserve. So far, there are four albumin-based liver reserve models specifically for liver cirrhosis and HCC. Our data demonstrate that the PALBI grade is not only an independent survival predictor in HCC patients undergoing TACE but also shows the best prognostic ability in terms of discriminating long-term survival in comparison with the other three albumin-based models and MELD scores. In addition, all four albumin-based models (PALBI, ALBI, EZ-ALBI and PAL grade) were better prognostic tools compared to the MELD for outcome prediction, suggesting the indispensable role of albumin in this clinical setting.

HCC typically develops in the background of chronic liver injury or cirrhosis. The rationale for adopting the four albumin-based models and MELD in HCC can also be demonstrated by the fact that their scores are higher (or less negative) in CTP class B than those in CTP class A (Figure 1).

The ALBI grade, based solely on serum albumin and bilirubin levels, is an objective tool to indicate liver functional reserve in HCC [14]. Consistent with previous studies [36,37,38,39], this study shows that the ALBI grade can discriminate long-term survival in HCC patients undergoing TACE. However, the calculation of the ALBI score is quite complex. Its updated version, the EZ-ALBI grade, is a more feasible method to estimate liver reserve and has been validated in the previous study [18]. The EZ-ALBI grade was shown to highly correlate with the ALBI grade, and it was consistently identified as an independent prognostic predictor in patients undergoing TACE in this study [18,19,40].

Although the ALBI and EZ-ALBI grades are useful methods to evaluate liver injury in HCC, the factor of portal hypertension is not considered in the formula. Notably, thrombocytopenia occurs as a result of hypersplenism in cirrhosis and is a surrogate marker for portal hypertension. Therefore, platelet count has been identified as an independent survival predictor in HCC [21,22,23,27,28]. The PALBI grade, based on serum albumin, bilirubin and platelet count, can well discriminate survival in patients undergoing TACE in our study. This result is consistent with previous studies [24,25,26]. Alternatively, the PAL score, based on platelet count and albumin level, can also well discriminate long-term outcomes in patients undergoing TACE, indicating this newly proposed marker is also a robust prognostic marker in HCC.

However, this study has some limitations. Firstly, this is a single-center study from a predominantly HBV-infected patient cohort. This feature is quite different from Western countries, where HCV infection and alcoholism are more prevalent. Secondly, this study was performed in patients undergoing TACE and the prognostic role of these models in other patient cohorts needs further study to be validated. Thirdly, treatment decisions were primarily based on the multidisciplinary HCC Board in our hospital and thus, they may not strictly adhere to the current Barcelona Clinic Liver Cancer (BCLC) recommendations.

## 5. Conclusions

Our study shows that the PALBI score is the best prognostic model in the setting of HCC patients undergoing TACE. All four albumin-based liver reserve models (PALBI, EZ-ALBI, PAL and ALBI) are better prognostic tools as compared with the MELD score. These data indicate the crucial role of albumin in the outcome prediction for HCC. External studies are required to confirm our findings.

## Figures and Tables

**Table 1:** Baseline characteristics of HCC patients undergoing transarterial chemoembolization (*n* = 1038).

Variables	*n* = 1038
Age (years, mean ± SD)	67 ± 13
Male/female, *n* (%)	786/252 (76/24)
Etiologies of liver disease	
HBV, *n* (%)	438 (42)
HCV, *n* (%)	309 (30)
HBV + HCV, *n* (%)	49 (5)
Others, *n* (%)	242 (23)
Performance status (0/1/2), *n* (%)	644/235/159 (62/23/15)
Tumor nodules (single/multiple)	547/491 (53/47)
Maximal tumor diameter ≥ 5 cm, *n* (%)	506 (49)
Tumor diameter, mean ± SD	6.3 ± 4.3
Vascular invasion, *n* (%)	185 (18)
Serum AFP (ng/mL), mean ± SD	18366 ± 131500
Serum AFP ≥ 200 ng/mL, *n* (%)	345 (33)
Ascites, *n* (%)	183 (18)
Laboratory values, mean ± SD	
Alanine transaminase (U/L)	65 ± 65
Albumin (g/dL)	3.7 ± 0.5
Total bilirubin (mg/dL)	1.1 ± 1.3
Platelets (10^4^/μL)	16.2 ± 9.4
INR of prothrombin time	1.08 ± 0.1
Creatinine (mg/dL)	1.1 ± 0.9
CTP score, mean ± SD	5.8 ± 1.0
CTP class (A/B), *n* (%)	823/215 (79/21)
ALBI score, mean ± SD	−2.33 ± 0.52
ALBI grade (1/2/3), *n* (%)	350/647/41 (34/62/4)
EZ-ALBI score, mean ± SD	−31.8 ± 5.2
EZ-ALBI grade (1/2/3), *n* (%)	352/646/40 (34/62/4)
MELD score, mean ± SD	9.1 ± 3.0
MELD score ≤8/>8, *n* (%)	420/618 (38/62)
PALBI score, mean ± SD	−2.38 ± 0.39
PALBI grade (1/2/3), *n* (%)	367/433/238 (35/42/23)
PAL score, mean ± SD	−3.5 ± 0.5
PAL grade (1/2/3), *n* (%)	317/537/184 (31/52/17)
BCLC stage (0/A/B/C), *n* (%)	32/196/288/552 (3/19/28/50)

ALBI, albumin-bilirubin; AFP, alpha-fetoprotein; BCLC, Barcelona Clinic Liver Cancer; CTP, Child–Turcotte–Pugh; EZ-ALBI, easy albumin-bilirubin, HBV, hepatitis B virus; HCV, hepatitis C virus; INR, international normalized ratio; MELD, model for end-stage liver disease; PALBI, platelet-albumin-bilirubin; PAL, platelet-albumin; SD, standard deviation.

**Table 2:** Formula and grading of noninvasive liver reserve models.

Noninvasive Liver Reserve Models	Formula
ALBI grade 1/2/3 (≤−2.6/>−2.6 and ≤−1.39/>−1.39)	(log_10_ (Bilirubin (μmol/L)) × 0.66) + (Albumin (g/L) × (−0.085))
EZ-ALBI grade 1/2/3 (≤−34.4, >−34.4 and ≤−22.2, >−22.2)	Bilirubin (mg/dL) − (9 × Albumin (g/dL))
PALBI grade 1/2/3 (≤−2.53), score > −2.53 and ≤−2.09)/(score > −2.09)	2.02 × log_10_ Bilirubin (μmol/L) level − 0.37 × (log_10_ Bilirubin level)^2^ − 0.04 × Albumin level − 3.48 × log_10_ Platelet count (1000/μL) + 1.01 × (log_10_ Platelet count)^2^
PAL grade 1/2/3 (score ≤ −3.77, score > −3.77 and ≤−3.04, score > −3.04	0.777 × Albumin (g/dL) − 0.575 × (log_10_ Platelet count) (10^4^/μL)
MELD, grade 1/2 (≤8/>8)	10 × (0.957 × ln(Creatinine)) + (0.378 × ln(Bilirubin (mg/dL))) + (1.12 × ln(INR)) + 6.43

ALBI, albumin-bilirubin; EZ-ALBI, easy albumin-bilirubin; PALBI, platelet-albumin-bilirubin; PAL, platelet-albumin; MELD, model for end-stage liver disease.

**Table 3:** Multivariate analysis of overall survival in patients undergoing transarterial chemoembolization including ALBI grade.

Overall Survival	Number	Univariate Analysis			Multivariate Analysis		
		HR	95% CI	*p*-Value	HR	95% CI	*p*-Value
Age (≤67/>67 years)	494/544	0.887	0.776–1.104	0.079			
Sex (male/female)	786/252	0.932	0.798–1.088	0.372			
HBV (negative/positive)	551/487	0.955	0.836–1.090	0.493			
HCV (negative/positive)	680/358	1.177	1.023–1.354	0.023			
Platelet (≥15/<15, 104/μL)	555/483	1.309	1.146–1.495	0.004			
Albumin (≥3.5/<3.5 g/dL)	682/356	1.414	1.230–1.626	<0.001			
Bilirubin (≤1.1/1.1 mg/dL)	707/331	0.935	0.811–1.078	0.355			
Creatinine (≤1.2/>1.2 mg/dL)	798/240	1.342	1.148–1.569	<0.001	1.267	1.082–1.483	0.003
Ascites (absent/present)	855/183	1.482	1.247–1.762	<0.001	1.262	1.055–1.510	0.011
Serum AFP (≤200/>200 ng/mL)	693/345	2.016	1.751–2.321	<0.001	1.745	1.507–2.020	<0.001
Vascular invasion (no/yes)	853/185	2.344	1.978–2.779	<0.001	1.621	1.354–1.941	<0.001
Tumor size (≤5 cm/>5 cm)	532/506	2.201	1.923–2.519	<0.001	1.863	1.610–2.155	<0.001
Tumor number (single/multiple)	547/491	0.965	0.844–1.102	0.595			
Performance status (0–1/2)	879/159	1.677	1.399–2.009	<0.001	1.227	1.015–1.484	0.034
ALBI grade 1/grade 2–3	352/686	1.556	1.349–1.796	<0.001	1.541	1.332–1.784	<0.001

**Table 4:** Multivariate analysis of overall survival in patients undergoing transarterial chemoembolization including EZ-ALBI grade.

Overall Survival	Number	Univariate Analysis			Multivariate Analysis		
		HR	95% CI	*p*-Value	HR	95% CI	*p*-Value
Age (≤67/>67 years)	494/544	0.887	0.776–1.104	0.079			
Sex (male/female)	786/252	0.932	0.798–1.088	0.372			
HBV (negative/positive)	551/487	0.955	0.836–1.090	0.493			
HCV (negative/positive)	680/358	1.177	1.023–1.354	0.023			
Platelet (≥15/<15, 104/μL)	555/483	1.309	1.146–1.495	0.004			
Albumin (≥3.5/<3.5 g/dL)	682/356	1.414	1.230–1.626	<0.001			
Bilirubin (≤1.1/1.1 mg/dL)	707/331	0.935	0.811–1.078	0.355			
Creatinine (≤1.2/>1.2 mg/dL)	798/240	1.342	1.148–1.569	<0.001	1.260	1.076–1.475	0.004
Ascites (absent/present)	855/183	1.482	1.247–1.762	<0.001			
Serum AFP (≤200/>200 ng/mL)	693/345	2.016	1.751–2.321	<0.001	1.729	1.493–2.002	<0.001
Vascular invasion (no/yes)	853/185	2.344	1.978–2.779	<0.001	1.613	1.347–1.931	<0.001
Tumor size (≤5 cm/>5 cm)	532/506	2.201	1.923–2.519	<0.001	1.853	1.602–2.143	<0.001
Tumor number (single/multiple)	547/491	0.965	0.844–1.102	0.595			
Performance status (0–1/2)	879/159	1.677	1.399–2.009	<0.001			
EZ-ALBI grade 1/grade 2–3	352/686	1.556	1.349–1.796	<0.001	1.563	1.350–1.810	<0.001

**Table 5:** Multivariate analysis of overall survival in patients undergoing transarterial chemoembolization including PALBI grade.

Overall Survival	Number	Univariate Analysis			Multivariate Analysis		
		HR	95% CI	*p*-Value	HR	95% CI	*p*-Value
Age (≤67/>67 years)	494/544	0.887	0.776–1.104	0.079			
Sex (male/female)	786/252	0.932	0.798–1.088	0.372			
HBV (negative/positive)	551/487	0.955	0.836–1.090	0.493			
HCV (negative/positive)	680/358	1.177	1.023–1.354	0.023			
Platelet (≥150/<150, 1000/μL)	555/483	1.309	1.146–1.495	0.004			
Albumin (≥3.5/<3.5 g/dL)	682/356	1.414	1.230–1.626	<0.001			
Bilirubin (≤1.1/1.1 mg/dL)	707/331	0.935	0.811–1.078	0.355			
Creatinine (≤1.2/>1.2 mg/dL)	798/240	1.342	1.148–1.569	<0.001	1.338	1.142–1.567	<0.001
Ascites (absence/present)	855/183	1.482	1.247–1.762	<0.001			
Serum AFP (≤200/>200 ng/mL)	693/345	2.016	1.751–2.321	<0.001	1.694	1.460–1.964	<0.001
Vascular invasion (no/yes)	853/185	2.344	1.978–2.779	<0.001	1.596	1.331–1.913	<0.001
Tumor size (≤5 cm/>5 cm)	532/506	2.201	1.923–2.519	<0.001	1.790	1.546–2.073	<0.001
Tumor number (single/multiple)	547/491	0.965	0.844–1.102	0.595			
Performance status (0–1/2)	879/159	1.677	1.399–2.009	<0.001			
PALBI grade 1/2–3	317/721	1.593	1.383–1.834	<0.001	1.358	1.163–1.584	<0.001

**Table 6:** Multivariate analysis of overall survival in patients undergoing transarterial chemoembolization including PAL grade.

Overall Survival	Number	Univariate Analysis			Multivariate Analysis		
		HR	95% CI	*p*-Value	HR	95% CI	*p*-Value
Age (≤67/>67 years)	494/544	0.887	0.776–1.104	0.079			
Sex (male/female)	786/252	0.932	0.798–1.088	0.372			
HBV (negative/positive)	551/487	0.955	0.836–1.090	0.493			
HCV (negative/positive)	680/358	1.177	1.023–1.354	0.023			
Platelet (≥15/<15, 104/μL)	555/483	1.309	1.146–1.495	0.004			
Albumin (≥3.5/<3.5 g/dL)	682/356	1.414	1.230–1.626	<0.001			
Bilirubin (≤1.1/1.1 mg/dL)	707/331	0.935	0.811–1.078	0.355			
Creatinine (≤1.2/>1.2 mg/dL)	798/240	1.342	1.148–1.569	<0.001	1.261	1.077–1.477	0.004
Ascites (absence/present)	855/183	1.482	1.247–1.762	<0.001			
Serum AFP (≤200/>200 ng/mL)	693/345	2.016	1.751–2.321	<0.001	1.732	1.496–2.005	<0.001
Vascular invasion (no/yes)	853/185	2.344	1.978–2.779	<0.001	1.663	1.389–1.991	<0.001
Tumor size (≤5 cm/>5 cm)	532/506	2.201	1.923–2.519	<0.001	1.949	1.683–2.257	<0.001
Tumor number (single/multiple)	547/491	0.965	0.844–1.102	0.595			
Performance status (0–1/2)	879/159	1.677	1.399–2.009	<0.001			
PAL grade 1/grade 2–3	352/686	1.556	1.349–1.796	<0.001	1.541	1.332–1.784	<0.001

**Table 7:** Multivariate analysis of overall survival in patients undergoing transarterial chemoembolization including MELD score.

Overall Survival	Number	Univariate Analysis			Multivariate Analysis		
		HR	95% CI	*p*-Value	HR	95% CI	*p*-Value
Age (≤67/>67 years)	494/544	0.887	0.776–1.104	0.079			
Sex (male/female)	786/252	0.932	0.798–1.088	0.372			
HBV (negative/positive)	551/487	0.955	0.836–1.090	0.493			
HCV (negative/positive)	680/358	1.177	1.023–1.354	0.023			
Platelet (≥15/<15, 104/μL)	555/483	1.309	1.146–1.495	0.004			
Albumin (≥3.5/<3.5 g/dL)	682/356	1.414	1.230–1.626	<0.001	1.451	1.256–1.677	<0.001
Bilirubin (≤1.1/1.1 mg/dL)	707/331	0.935	0.811–1.078	0.355			
Creatinine (≤1.2/>1.2 mg/dL)	798/240	1.342	1.148–1.569	<0.001	1.270	1.085–1.487	0.003
Ascites (absence/present)	855/183	1.482	1.247–1.762	<0.001			
Serum AFP (≤200/>200 ng/mL)	693/345	2.016	1.751–2.321	<0.001	1.709	1.476–1.979	<0.001
Vascular invasion (no/yes)	853/185	2.344	1.978–2.779	<0.001	1.673	1.397–2.003	<0.001
Tumor size (≤5/>5 cm)	532/506	2.201	1.923–2.519	<0.001	1.855	1.603–2.146	<0.001
Tumor number (single/multiple)	547/491	0.965	0.844–1.102	0.595			
Performance status (0–1/2)	879/159	1.677	1.399–2.009	<0.001			
MELD score ≤8/>8	420/618	1.176	1.027–1.346	0.019			

**Table 8:** Comparison of non-invasive liver reserve model in hepatocellular carcinoma patients undergoing transarterial chemoembolization (*n* = 1038).

Non-Invasive Liver Reserve Model	Homogeneity (Wald χ^2^)	AICc
ALBI	26.009	10,782.486
EZ-ALBI	34.862	10,773.633
PALBI	55.041	10,753.453
PAL	31.427	10,777.061
MELD	5.550	10,802.944

## Data Availability

The data presented in this study are available in this article.
